# Fungemia caused by *Cyberlindnera fabianii*: Clinical and microbiological insights from two fatal cases

**DOI:** 10.1016/j.mmcr.2026.100813

**Published:** 2026-07-18

**Authors:** Michał Sarzyński, Maria Łuczak, Adrian Bekier, Eliza Miaśkiewicz, Dorota Pastuszak-Lewandoska, Małgorzata Brauncajs

**Affiliations:** a Department of Microbiology and Medical Laboratory Immunology, Medical University of Lodz, Łódź, 92-213, Poland; b Laboratory of Medical Microbiology, Central Teaching Hospital of Medical University of Lodz, Łódź, 92-213, Poland

**Keywords:** *Cyberlindnera fabianii* fungemia, Opportunistic yeast, Antifungal susceptibility, Case report

## Abstract

*Cyberlindnera fabianii* is a rare opportunistic yeast increasingly recognized as a cause of invasive infections. We report two fatal cases of *C. fabianii* fungemia in elderly patients with multiple comorbidities. Microbiological analysis revealed low minimum inhibitory concentrations (MICs) to amphotericin B and echinocandins, but variable susceptibility to fluconazole. One patient received antifungal therapy with limited response; in the second case, the pathogen was identified postmortem.

## 1 Introduction

Fungemia is a major threat to hospitalized patients, especially those who are immunocompromised, neutropenic, oncology patients, or individuals with invasive vascular devices [1]. Although Candida species remain the leading cause of fungal bloodstream infections, an increasing number of cases are attributed to rare opportunistic yeasts, which are often difficult to diagnose and treat [2,3].

*Cyberlindnera fabianii* (formerly *Pichia fabianii*, *Lindnera fabianii*, and *Candida fabianii*) is a rare opportunistic yeast from the Phaffomycetaceae family [4], typically found in environmental sources such as fermented products and industrial waste. First described in 1964, it appears microscopically (measuring 3.0–6.5 × 2.0–5.5 μm in size) as budding spheroidal to ellipsoidal cells and forms smooth, cream-colored colonies on Sabouraud dextrose agar (SDA) [5].

One of the main challenges in managing *C. fabianii* infections is achieving accurate identification of the pathogen. Standard phenotypic identification methods (such as API 20C AUX or VITEK yeast biochemical panels) frequently misclassify this yeast as other species – for example, as *Cyberlindnera jadinii* or *Wickerhamomyces anomalus* due to overlapping metabolic profiles [6,7]. In one analysis, a high proportion of isolates initially identified biochemically as *W. anomalus* or *C. jadinii* were later confirmed to be *C. fabianii* upon molecular analysis. Such misidentification can lead to inappropriate management. Therefore, the use of advanced identification techniques is increasingly recommended to definitively identify *C. fabianii* at the species level [6,8]. DNA sequencing of the ribosomal ITS1-2 region and D1/D2 domain, or of the EF-1α gene, provides precise identification and can distinguish *C. fabianii* from closely related yeasts [6,8]. Likewise, matrix-assisted laser desorption/ionization time-of-flight mass spectrometry (MALDI-TOF MS) has demonstrated strong discriminatory power for identifying *C. fabianii* when updated spectral databases are used.

Due to the limited number of cases, no species-specific treatment guidelines exist for *C. fabianii*. Reported infections have been treated with fluconazole, echinocandins, and amphotericin B, with variable outcomes. A key challenge is its ability to form biofilms on medical devices, which reduces antifungal susceptibility. Biofilm-associated cells show marked resistance to fluconazole (MICs ≥512 μg/mL), whereas planktonic cells are more susceptible [9]. In contrast, echinocandins and amphotericin B demonstrate better efficacy against both planktonic and biofilm forms [9]. Clinical reports support improved outcomes after switching to these agents, particularly when combined with removal of infected devices [6,10]. These findings suggest that echinocandins or amphotericin B may be preferable as first-line therapy when *C. fabianii* infection is suspected. The ability of *C. fabianii* to rapidly acquire resistance to azole antifungals has already been noted, underscoring the need for ongoing surveillance and antifungal susceptibility testing [11].

## 2 Case presentation

### 2.1 Case report 1

1. Patient Presentation and Initial Assessment

A 74-year-old woman with chronic heart failure and acute kidney injury was admitted with life-threatening anemia (hemoglobin 3.1 g/dL) and severe abdominal pain. On exam she was in poor general condition: malnourished, disheveled, disoriented, and hypotensive, but afebrile. Initial assessment revealed a markedly distended urinary bladder; catheterization drained ∼1 L urine and partially relieved her symptoms. Given her decompensated state, she was admitted to nephrology for intensive management (day 0).

2. Diagnostic workup

Gastrointestinal endoscopy identified erosive gastritis, a duodenal ulcer (no active bleed) and a bleeding gastric erosion, which was endoscopically coagulated.

**Fig. 1:**
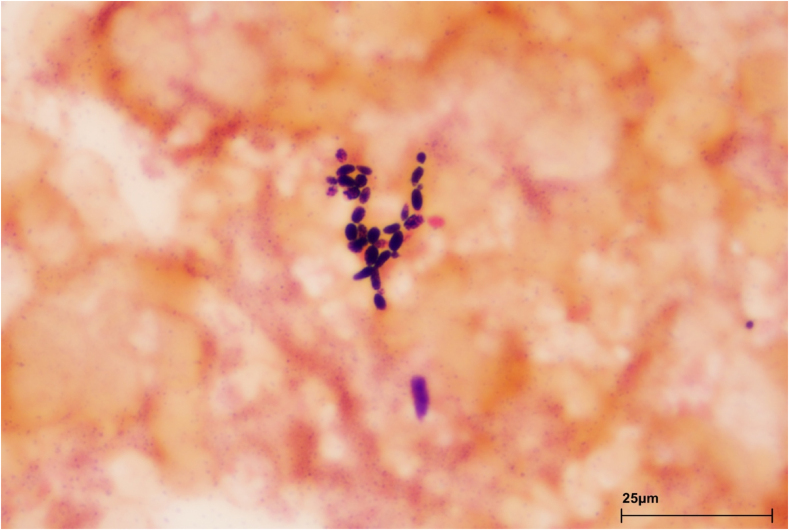
Microscope view of gram-stained blood described in the report (original magnification ×1000).

During hospitalization, progressive clinical deterioration became evident and, on day 11, urine and blood cultures were obtained because of increasing inflammatory markers, respiratory symptoms (dyspnea and oxygen desaturation requiring supplemental oxygen therapy), pulmonary infiltrates on imaging studies, and hemodynamic instability requiring vasopressor support. The patient also had an indwelling urinary catheter and clinical features suggestive of a urinary tract infection. These findings raised suspicion of a systemic infectious process and prompted microbiological investigations. Blood cultures later revealed a central line–associated bloodstream infection due to *Staphylococcus haemolyticus*, leading to modification of the antibiotic regimen. Following targeted antimicrobial therapy, the patient's clinical condition and inflammatory markers initially improved. However, on day 20, her condition deteriorated again, prompting collection of additional blood samples for culture from two new independent punctures on the upper limbs. After 35 h of incubation in the BACT/ALERT® VIRTUO® system (bioMérieux, France), the aerobic bottles were flagged as positive. Due to the patient's critical condition, a syndromic BIOFIRE® Blood Culture Identification 2 (BCID2) panel was performed, but the test result was negative. In addition, a Gram-stained microscopic preparation revealed blastospores (Fig. 1).

**Fig. 2:**
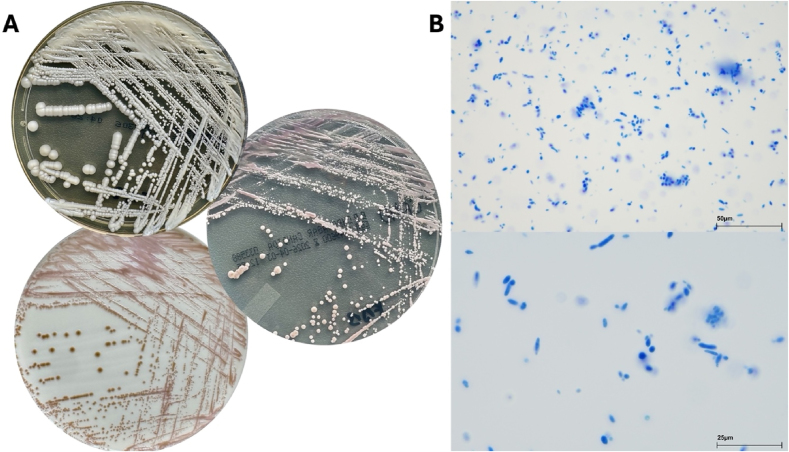
Macroscopic and microscopic examinations of *Cyberlindnera fabianii* from a patient. **A.** Macroscopic aspect of *C. fabianii* on three agar media: Sabouraud dextrose agar with chloramphenicol and gentamicin after incubation for 96 h at 30°C (upper left); BD BBL™ CHROMagar™ Candida for 96 h at 35°C (middle); Brilliance™ Candida agar after incubation for 96 h at 35°C (lower left). **B***. C fabianii* from Sabouraud agar in BactiDrop™ Lactophenol Aniline Blue (Remel), showing spherical to ellipsoidal yeast cells with budding and pseudohyphae (original magnification ×400 and ×1000).

The positive blood sample was cultivated on Sabouraud dextrose agar with chloramphenicol and gentamicin (OXOID, UK) and incubated at 30°C. After 48h the colonies appeared shiny and cream-colored. Growth on chromagar, Brilliance™ Candida agar (OXOID, UK) and BD BBL™ CHROMagar™ Candida (Becton Dickinson, USA), were slower and produced smaller colonies compared to Sabouraud, with a brown or pink-cream coloration, respectively. On the microscopy, yeast cells were spherical to ellipsoidal and had budding and form pseudohyphae (Fig. 2). The strains were identified using matrix-assisted laser desorption/ionization time-of-flight mass spectrometry (MALDI-TOF MS), which classified the organism as *Candida fabianii*, and further confirmed by IndexFungorum as *Cyberlindnera fabianii*. Identification was carried out with the VITEK® MS system (bioMérieux, France).

No specific breakpoints have been established for the antifungal susceptibility of *Cyberlindnera* spp., but we referred to the EUCAST document Interpretation of MICs for Rare Yeast without Breakpoints in Breakpoint Tables (version 2024-06-19), which provided the basis for interpreting susceptibility to four antifungal agents: amphotericin B (MIC ≤1), fluconazole (MIC ≤2), voriconazole (MIC ≤0.03), and anidulafungin (MIC ≤0.06). Fungal isolates were tested using antifungal gradient strips (Liofilchem, USA). Suspensions were adjusted to 0.5 McFarland in 0.85% NaCl and inoculated onto RPMI 1640 agar with MOPS and 2% glucose (Liofilchem, USA). Gradient strips were applied 15 min post-inoculation. Plates were incubated at 35°C with increased humidity for 24 and 48 h. MICs were determined manually at both time points. Moreover, antifungal susceptibility testing was also performed according to CLSI method using Sensititre™ YeastOne™ YO10 panel (Thermo Scientific™, USA). In Table 1 we summarized antifungal susceptibility of both methods.

**Table 1:** Antifungal susceptibility profile of *Cyberlindnera fabianii* isolated from blood culture in Case 1.

Antifungal agent	Liofilchem gradient strips (mg/L)		Sensititre™ YeastOne™ YO10 panel (mg/L)
	24h	48h	
5-Flucytosine	-	-	2.00
Amphotericin B	0.03	0.125	0.25
Anidulafungin	0.008	0.016	0.015
Micafungin	-	-	0.015
Caspofungin	-	-	0.03
Fluconazole	0.50	1.00	0.50
Voriconazole	0.016	0.016	0.015
Itraconazole	0.064	0.125	0.015
Posaconazole	0.125	0.25	0.03

There are no established treatment guidelines for *C. fabianii*. Therefore, the microbiological test report was accompanied by the following comment based on the EUCAST guidance: Formal categorizing of the susceptibility of the organism is not possible. A cautious interpretation suggests that the amphotericin B or anidulafungin may be considered for therapy.

3. Treatment and clinical course

Hemodynamic instability required continuous norepinephrine infusion and cautious IV fluids. She remained anemic/coagulopathic despite transfusions; repeated endoscopies were needed to identify and coagulatively treat a bleeding esophageal ulcer. Hemostatic agents (tranexamic acid, ethamsylate) and vitamin K were given. Initial empiric antibiotics (amoxicillin/clavulanate) were broadened to include levofloxacin and piperacillin–tazobactam for presumed urinary and respiratory infections. Once *S. haemolyticus* was identified in blood cultures, therapy was tailored accordingly to vancomycin.

For the fungal infection, fluconazole was used first, loading dose 800 mg, then 400 mg/day intravenously, for 5 days, then switched to voriconazole when fever and inflammatory markers persisted 6 mg/kg twice daily on day 1, followed by 4 mg/kg twice daily intravenously which was continued for 10 days. The isolate's MIC profile (particularly its very low voriconazole MIC) was consistent with a good, expected response.

At the time of recurrent clinical deterioration that prompted repeat blood cultures (day 20), laboratory findings showed leukocytosis (14.32 G/L), elevated inflammatory markers (CRP 113.9 mg/L), and a marked increase in procalcitonin concentration (65.7 μg/L). Blood cultures obtained from two independent peripheral venipunctures subsequently yielded *C. fabianii*. Following initiation of antifungal therapy, inflammatory markers gradually decreased, accompanied by reductions in leukocyte count (14.32 to 8.22 G/L) and procalcitonin concentration (65.7 to 1.5 μg/L). These findings suggest that *C. fabianii* fungemia may have contributed to the patient's infectious burden, although its precise role in the overall clinical course remains difficult to determine because of the patient's complex condition, including gastrointestinal bleeding, hemorrhagic shock, and preceding *S. haemolyticus* bloodstream infection.

Despite maximal supportive efforts, the patient developed progressive organ failure. She required escalating vasopressors for circulatory collapse, and mechanical ventilatory support for respiratory failure. Renal function remained impaired. Serial imaging and labs guided care, but the patient's condition remained critical.

4. Outcome

On the final day of hospitalization, the patient experienced sudden clinical deterioration with cardiac arrest due to asystole. Due to the lack of response to resuscitation and a poor prognosis, the decision was made not to pursue further resuscitative efforts. The patient was pronounced dead (day 48).

### 2.2 Case report 2

1. Patient Presentation and Initial Assessment

An 83-year-old woman was urgently admitted to the Nephrology, Hypertension, and Kidney Transplant Unit after emergency medical services found her lying on the floor at home, unable to call for help. However, she had marked pitting edema of both lower limbs and clinical signs suggesting a urinary tract infection. Her medical history was notable for a known neoplastic lesion of the left kidney, renal tumor.

2. Diagnostic workup

Initial laboratory tests showed evidence of systemic inflammation (C-reactive protein 29.5 mg/L, procalcitonin 0.68 μg/L), leukocytosis (WBC 13.0×10^∧^3/μL with neutrophilia) and elevated muscle enzymes consistent with rhabdomyolysis. Urinalysis demonstrated cloudy, red urine with abundant bacteriuria, leukocyturia, and hematuria. Abdominal imaging (ultrasound) confirmed the left renal mass (approximately 96 × 75 mm) without clear hydronephrosis; the right kidney was unremarkable. Based on the clinical signs of urinary tract infection, urine cultures were obtained before initiation of antimicrobial therapy. No blood cultures were collected at that stage of hospitalization.

3. Treatment and clinical course

Empirical antibiotics (intravenous amoxicillin–clavulanate) were started for presumed urosepsis, along with aggressive intravenous fluids and hemostatic support due to hematuria. On hospital day 2, cultures grew *Escherichia coli*. In response to persistent fever and rising inflammatory markers, the regimen was changed to ceftriaxone plus ciprofloxacin (double Gram-negative coverage). By day 9, a new increase in inflammatory markers and clinical deterioration prompted a diagnosis of hospital-acquired pneumonia; antibiotic therapy was escalated to vancomycin and meropenem.

During her stay she had ongoing hematuria with anemia. She required transfusion of 2 units of packed red cells (Hb had fallen to 7.5 g/dL). Diuretics and fluid restriction were used to manage her edema and rhabdomyolysis, which improved renal function.

On hospital day 10, the patient acutely deteriorated: her blood pressure first spiked to ∼220/100 mmHg and then precipitously fell despite escalating vasopressors. The right thigh became slightly swollen but non‐tender. Laboratory tests on that day showed worsening anemia (Hb 6.3 g/dL). Hours later, a large ecchymosis and rapidly enlarging hematoma appeared over the medial right thigh.

On day 11, CT angiography of the thigh revealed a massive hematoma in the medial compartment of the right thigh with active bleeding from branches of the profunda femoris artery. Vascular surgery was consulted twice; initially no operation was recommended, but as bleeding persisted the patient was taken emergently to the operating room. Under general anesthesia, the hematoma was evacuated and hemostasis was achieved; two drains were placed.

Postoperatively she remained critically ill, intubated and on mechanical ventilation in the ICU. Multiorgan failure and refractory metabolic acidosis ensued despite full support. Blood cultures were obtained within a few hours after ICU admission on hospital day 11. These were the first blood cultures collected during the patient's hospitalization. The cultures later yielded a yeast. Despite maximal therapy, the patient suffered cardiac arrest (asystole) the next morning and resuscitation was unsuccessful.

4. Outcome

Postmortem review of microbiology revealed *Cyberlindnera fabianii* in blood cultures obtained shortly after ICU admission. Species identification was performed using matrix-assisted laser desorption/ionization time-of-flight mass spectrometry (MALDI-TOF MS), initially classifying the isolate as *Candida fabianii*. Subsequent verification via the IndexFungorum taxonomy database confirmed its current nomenclature as *Cyberlindnera fabianii*. The analysis was conducted using the VITEK® MS platform (bioMérieux, France).

Antifungal susceptibility testing was performed using two complementary methods. First, gradient diffusion assays (Liofilchem, USA) were conducted on RPMI 1640 agar supplemented with MOPS and 2% glucose. Fungal suspensions were standardized to 0.5 McFarland in 0.85% NaCl, and gradient strips were applied 15 min after inoculation. Plates were incubated at 35°C under humidified conditions, and minimum inhibitory concentrations (MICs) were read manually after 24 and 48 h. Additionally, broth microdilution testing was carried out according to CLSI guidelines using the Sensititre™ YeastOne™ YO10 panel (Thermo Scientific™, USA). Table 2 summarizes the MIC results obtained with both methodologies.

**Table 2:** Antifungal susceptibility profile of *Cyberlindnera fabianii* isolated from blood culture in Case 2.

Antifungal agent	Liofilchem gradient strips (mg/L)		Sensititre™ YeastOne™ YO10 panel (mg/L)
	24h	48h	
5-Flucytosine	-	-	0.125
Amphotericin B	0.125	0.25	0.25
Anidulafungin	0.03	0.06	0.03
Micafungin	-	-	0.015
Caspofungin	-	-	0.125
Fluconazole	1.00	2.00	1.00
Voriconazole	0.016	0.03	0.015
Itraconazole	0.064	0.125	0.06
Posaconazole	0.125	0.25	0.03

There are no established treatment guidelines for *C. fabianii*. Therefore, the microbiological test report was accompanied by the following comment based on the EUCAST guidance: Formal categorizing of the susceptibility of the organism is not possible. A cautious interpretation suggests that the amphotericin B or anidulafungin may be considered for therapy.

Unfortunately, the identification of this yeast arrived after the patient's demise, so no directed antifungal therapy was given. In retrospect, the in vitro data suggest that treatment with an appropriate antifungal (e.g. fluconazole or an echinocandin) would likely have been effective, had the infection been recognized and treated earlier.

## 3 Discussion

Both patients had typical risk factors for opportunistic fungal infection: advanced age, multiple comorbidities, prolonged hospitalization, broad-spectrum antibiotics, metabolic disturbances, and recent severe bacterial infections. These conditions, along with immunosuppression and intensive care support, likely facilitated fungal invasion.

A key factor in both cases was long-term central venous access. The organism's ability to form biofilms on plastic surfaces makes catheter colonization a likely source of infection. Current guidelines recommend prompt catheter removal for source control, as this improves antifungal treatment outcomes [1]. In our cases, catheters were retained due to patient instability, which may have contributed to persistent infection.

Despite its low virulence, *C. fabianii* can cause invasive infections, including fungemia, in high-risk patients. More than 20 cases have been reported, mainly in neonates and immunocompromised adults [[10], [11], [12], [13], [14], [15], [16], [17], [18], [19]]. Infections are frequently associated with medical interventions such as central venous catheters, mechanical ventilation, or parenteral nutrition. A neonatal outbreak highlights its potential for nosocomial spread. To place our findings in context, we reviewed published cases of *C. fabianii* fungemia reported in the literature (Table 3).

**Table 3:** Published cases of *Cyberlindnera fabianii* fungemia reported in the literature.

No.	Year	Country	Patient Description	Risk Factors	Treatment	Outcome	Source
1	2015	Spain	2-year-old boy with short bowel syndrome	Malnutrition, antibiotic therapy	Caspofungin	Recovery	[12]
2	2016	Japan	69-year-old woman with acute mixed-phenotype leukemia after transplant	Immunosuppression, transplantation	Not specified	Death	[13]
3	2013	China	Premature infant with low birth weight	Prematurity, mechanical ventilation	Fluconazole	Recovery	[14]
4	2019	India	Premature twins	Prematurity, NICU	Fluconazole	Deaths	[15]
5	2015	South Korea	Patient with lung cancer and pulmonary infection	Immunosuppression, mechanical ventilation	Anidulafungin	Recovery (no fungus in blood), but died from other cause	[16]
6	2023	South Korea	Patient with vascular port infection	Vascular port	Micafungin	Recovery	[17]
7	2012	France	Patient after mesenteric ischemia	Surgical intervention	Caspofungin	Recovery	[18]
8	2019	USA	37-year-old man with B-cell lymphoma	Rituximab, immunosuppression	Micafungin, Voriconazole, Amphotericin B	Death	[11]
9	2008	Croatia	3.5-year-old girl with leukemia	Leukemia, neutropenia, antibacterial therapy	Fluconazole, Amphotericin B	Recovery	[19]
10	2012	Croatia	Newborn with intestinal atresia	Intestinal atresia, surgery, parenteral nutrition, antibiotics	Fluconazole	Recovery	[19]
11	2012	Croatia	Premature infant with low birth weight	Prematurity, lung cyst, 740 g weight, parenteral nutrition, antibacterial therapy	Fluconazole, Caspofungin	Recovery	[19]
12	N/A	Kuwait	Neonate/M, fungemia	No data	Fluconazole, Amphotericin B, Caspofungin	Recovery	[10]
13	N/A	Kuwait	Neonate/F, fungemia	No data	None	Death	[10]
14	N/A	Kuwait	Neonate/M, fungemia	No data	Amphotericin B, Fluconazole	Recovery	[10]
15	N/A	Kuwait	Neonate/F, fungemia	No data	Amphotericin B, Caspofungin	Recovery	[10]
16	N/A	Kuwait	Neonate/F, fungemia	No data	Amphotericin B, Caspofungin	Recovery	[10]
17	N/A	Kuwait	Neonate/M, fungemia	No data	Amphotericin B	Recovery	[10]
18	N/A	Kuwait	Neonate/M, fungemia	No data	Amphotericin B	Recovery	[10]
19	N/A	Kuwait	Neonate/F, fungemia	No data	Amphotericin B, Fluconazole, Caspofungin	Recovery	[10]
20	N/A	Kuwait	Neonate/F, fungemia	No data	Amphotericin B, Fluconazole, Caspofungin	Recovery	[10]
21	N/A	Kuwait	Neonate/F, fungemia	No data	Amphotericin B	Recovery	[10]
22	N/A	Kuwait	Neonate/F, fungemia	No data	Amphotericin B, Fluconazole, Caspofungin	Recovery	[10]

Review of the published literature demonstrates that *C. fabianii* fungemia predominantly affects highly vulnerable patients, including premature neonates, individuals with hematological malignancies, transplant recipients, and critically ill adults. Across reported cases, the most common predisposing factors include broad-spectrum antibiotic exposure, central venous catheterization, intensive care unit stay, parenteral nutrition, and severe underlying disease. Both patients described in our report shared several of these recognized risk factors, particularly advanced age, prolonged hospitalization, multiple comorbidities, exposure to broad-spectrum antimicrobial therapy, and the presence of intravascular devices.

The occurrence of two cases of *C. fabianii* fungemia within a relatively short period prompted consideration of possible nosocomial transmission. However, no epidemiological association between the cases was identified. The infections occurred approximately three months apart, involved patients hospitalized in different wards, and no common healthcare personnel, procedures, or other potential transmission routes could be established. Therefore, horizontal transmission within the hospital was considered unlikely.

There are no species-specific treatment guidelines for *C. fabianii* due to its rarity. Reported cases have been treated with various antifungals, but evidence suggests that echinocandins and amphotericin B are more reliable than azoles, partly due to reduced azole susceptibility in biofilms. For example, mature *C. fabianii* biofilms have fluconazole MICs ≥512 mg/L, whereas amphotericin B and echinocandins retain low MICs even against sessile cells [9]. Clinically, many patients improve only after switching from an azole to an echinocandin or liposomal amphotericin B, often alongside device removal. In line with this, one series from Korea found that voriconazole and echinocandins showed good activity against most *C. fabianii* isolates [20].

We compared our isolates' MICs to those in the literature. In the study by Tóth et al. (2022), eight clinical *C. fabianii* isolates were tested [9]. Planktonic MIC ranges reported were fluconazole 1–2 mg/L, amphotericin B 0.25–1 mg/L, anidulafungin 0.015–0.06 mg/L, caspofungin 0.03–0.12 mg/L, and micafungin 0.25–0.5 mg/L. One-day-old biofilms of these strains showed MICs ≥512 mg/L for fluconazole, while amphotericin B (0.25–2 mg/L) and echinocandins (0.06–2 mg/L) remained active. By comparison, the MICs we obtained were at the low end of these ranges. Notably, our micafungin MIC (0.015 mg/L) was at or below the lowest value reported, suggesting very high susceptibility. Our fluconazole MICs (0.5–1 mg/L) were also below those in Tóth et al., although these results apply to planktonic cells only. In one published case (Lee et al., 2015) [16], the isolate's anidulafungin MIC was 0.03 mg/L and fluconazole 2 mg/L comparable to or higher than ours. Taken together, these data indicate that our isolates were at least as susceptible as typical strains. Other reports, including Korean surveillance studies, similarly show that echinocandins and voriconazole have the lowest MICs against *C. fabianii*. In summary, our findings confirm that echinocandins and amphotericin B tend to have the greatest in vitro activity, while azoles especially fluconazole often face higher MICs, an observation that is especially concerning if biofilms are present.

In conclusion, *C. fabianii* should be recognized as an emerging opportunistic yeast capable of causing invasive bloodstream infections in critically ill and highly comorbid patients. Accurate identification using MALDI-TOF MS and awareness of this uncommon pathogen are essential, as routine syndromic diagnostic panels may fail to detect it. Although standardized treatment recommendations are lacking, our findings and the available literature support the use of echinocandins or amphotericin B as the most reliable therapeutic options. Increased recognition and reporting of *C. fabianii* infections are needed to improve understanding of its epidemiology, antifungal susceptibility patterns, and optimal clinical management.

## CRediT authorship contribution statement

**Michał Sarzyński:** Formal analysis, Writing – original draft. **Maria Łuczak:** Formal analysis, Writing – original draft. **Adrian Bekier:** Conceptualization, Data curation, Formal analysis, Funding acquisition, Investigation, Methodology, Project administration, Resources, Supervision, Validation, Visualization, Writing – original draft, Writing – review & editing. **Eliza Miaśkiewicz:** Formal analysis, Investigation, Methodology, Writing – original draft. **Dorota Pastuszak-Lewandoska:** Writing – original draft, Writing – review & editing. **Małgorzata Brauncajs:** Conceptualization, Data curation, Formal analysis, Funding acquisition, Investigation, Methodology, Project administration, Resources, Supervision, Validation, Writing – original draft, Writing – review & editing.
